# Digital Twin-Based Fault Diagnosis Platform for Final Rolling Temperature in Hot Strip Production

**DOI:** 10.3390/ma16217021

**Published:** 2023-11-03

**Authors:** Chen Desheng, Shao Jian, Li Mingxin, Xiang Sensen

**Affiliations:** National Engineering Research Center of Flat Rolling Equipment, University of Science and Technology Beijing, Beijing 100083, China; m202121225@xs.ustb.edu.cn (C.D.); a983271598@gmail.com (L.M.); m202321384@xs.ustb.edu.cn (X.S.)

**Keywords:** final rolling temperature, digital twin, knowledge graph, hot-rolled strip, fault diagnosis

## Abstract

The final rolling temperature in hot rolling is an important process parameter for hot-rolled strips and greatly influences their mechanical properties and rolling stability. The diagnosis of final rolling temperature anomalies in hot rolling has always been difficult in industry. A data-driven risk assessment method for detecting final rolling temperature anomalies is proposed. In view of the abnormal setting value for the strip head, a random forest model is established to screen the process parameters with high feature importance, and the isolation forest algorithm is used to evaluate the risk associated with the remaining parameters. In view of the abnormal process curve of the full length of the strip, the Hausdorff distance algorithm is used to eliminate samples with large deviations, and a risk assessment of the curve is carried out using the *LCSS* algorithm. Aiming to understand the complex coupling relationship between the influencing factors, a method for identifying the causes of anomalies, combining a knowledge graph and a Bayesian network, is established. According to the results of the strip head and the full-length risk assessment model, the occurrence of the corresponding nodes in the Bayesian network is determined, and the root cause of the abnormality is finally output. By combining mechanistic modeling and data modeling techniques, it becomes possible to rapidly, automatically, and accurately detect and analyze final rolling temperature anomalies during the rolling process. When applying the system in the field, when compared to manual analysis by onsite personnel, the accuracy of deducing the causes of anomalies was found to reach 92%.

## 1. Introduction

With the progress and development of the country, the demand for steel products in all walks of life continues to increase; simultaneously, the requirements for steel quality continue to rise [1]. The final rolling temperature is the temperature measured when the hot-rolled strip leaves the last finishing mill. It is an important process parameter in the hot-rolling process, which has a great impact on the mechanical properties of the strip [2], the thickness of the strip [3], and the rolling stability [4]; therefore, it is very important for the abnormal diagnosis of the final rolling temperature.

There are currently three main approaches to the diagnosis of abnormalities in finishing rolling temperatures, namely diagnosis based on expert experience, diagnosis based on mathematical models, and diagnosis based on data-driven methods [5,6,7]. (1) Expert experience-based diagnostic method: In manufacturing environments, this method is commonly employed for analyzing the causes of faults in strip steel production. Technical personnel rely on their own experience to accurately pinpoint faults and analyze their root causes based on relevant key process parameters and on-site production conditions. For instance, Bahrami et al. observed surface cracks in C-Mn structural steel through manual examination and found that surface cracks were caused by high oxygen content during the preheating stage, leading to oxidation within the V-shaped grooves on the surface [8]. However, relying solely on expert experience for diagnosis has issues such as subjectivity, low diagnostic efficiency, and challenges in achieving high-precision quality control. (2) Mathematical model-based diagnostic method: The mathematical model-based approach involves modeling the hot-rolling production process using physical or mathematical models. Due to the support of specific theoretical models, this method can provide more accurate predictions and simulations of variations in parameters during production. For example, Li et al. proposed a novel elastic-plastic multistand FE (Finite Element) model for the TCR (Tandem Cold Rolling) process that considered the work hardening effect, utilizing a segmentation modeling strategy, data transfer technologies, and element remesh technology. This model was used to quantitatively investigate the effects of rolling force on strip crown and flatness at each stand [9]. However, this modeling process demands high accuracy for parameter values, is complex, and suffers from poor model transferability. (3) Data-driven diagnostic method: Data-driven approaches involve collecting extensive data samples; preprocessing the data through filtering, normalization, noise reduction, etc.; and using machine learning algorithms to establish data models. These models are trained on collected samples or used for data mining and analysis to uncover relationships and features within the data. For example, Sui X. and Lv Z. utilized a feature subset extraction algorithm combining information entropy and Gram–Schmidt orthogonal transformation in their research on predicting the performance of hot-rolled products. They extracted key influencing factors for the finishing rolling temperature, providing insights into the root causes of anomalies in hot-rolled strip steel [10]. Data-driven methods have the advantage of not relying on subjective human judgment, and adapt well to different datasets, but their diagnostic accuracy is highly influenced by the quality of the dataset. After summarizing previous research, it is noted that few scholars have combined the above methods to address these issues, especially the integration of on-site expert experience, mechanistic models, and data-driven algorithms.

The method currently used to diagnose abnormalities in the final rolling temperature in steel mills is analyzing the process data manually and then feeding back the results to the production line for adjustment. There are many influencing factors in the production process, and there is a coupling relationship between the process parameters. Therefore, the efficiency of manual analysis is low, and the technical staff’s own professional knowledge is required. In order to solve the above problems, this study adopted a hybrid modeling method and data and expert experience to diagnose abnormal situations regarding the finishing rolling temperature. The hybrid modeling method can combine the advantages of the three, including the accuracy of the mechanism model and the data-driven efficiency and adaptability, and also combines the valuable manual experience on site to further modify the model. The research process was divided into the following three steps. First, a twin-based fault diagnosis platform for a hot-rolling production line was established, accessing the required data through the platform. Second, the strip steel was divided into the head and the body, and different methods were adopted for different parts to obtain the greatest influencing factors. Finally, the knowledge graph method combined with the Bayesian algorithm was used to build a database of field expert experience to automatically obtain the root cause of the abnormal final rolling temperature of hot-rolled strips.

## 2. Experimental Procedure

### 2.1. Fault Diagnosis Platform for Hot-Rolled Strip Based on Digital Twin

Digital twin technology can realize the interconnection and deep integration of the physical world and the virtual world. It possesses the capability to predict and control product states in the monitoring of complex product manufacturing processes [11].

**Figure 1:**
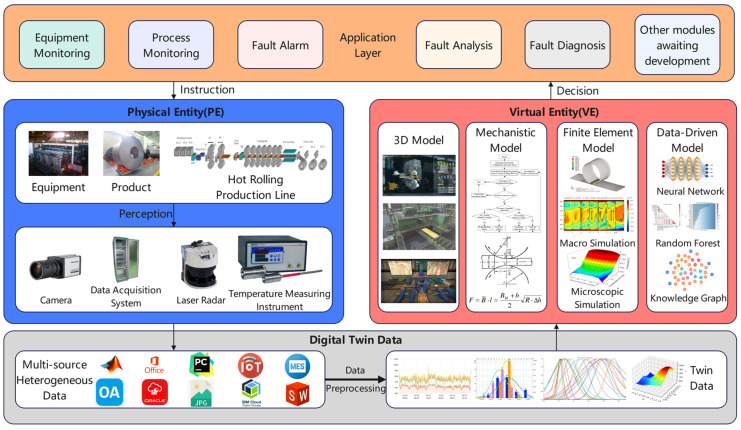
Digital twin-based hot-rolled strip steel fault diagnosis platform.

The fault diagnosis of process parameters involves multiple equipment and production lines; how to integrate the knowledge of different processes and different mechanisms is a key technical difficulty. In this study, the fault diagnosis of process parameters is combined with digital twin technology to build a fault diagnosis platform for hot-rolled strip steel [12], which includes an application layer, an edge layer, an entity, a sensing device, and a twin, as shown in Figure 1.

#### 2.1.1. Entity Perception Layer

The physical entity is the foundation and service object of the system, and the system finally gives the entity awareness layer a better decision deployment. The physical sensing layer includes hot-rolling equipment, hot-rolled products, hot-rolling production lines, and various sensing sensors, which can provide production process data.

#### 2.1.2. Digital Twin Data

**Figure 2:**
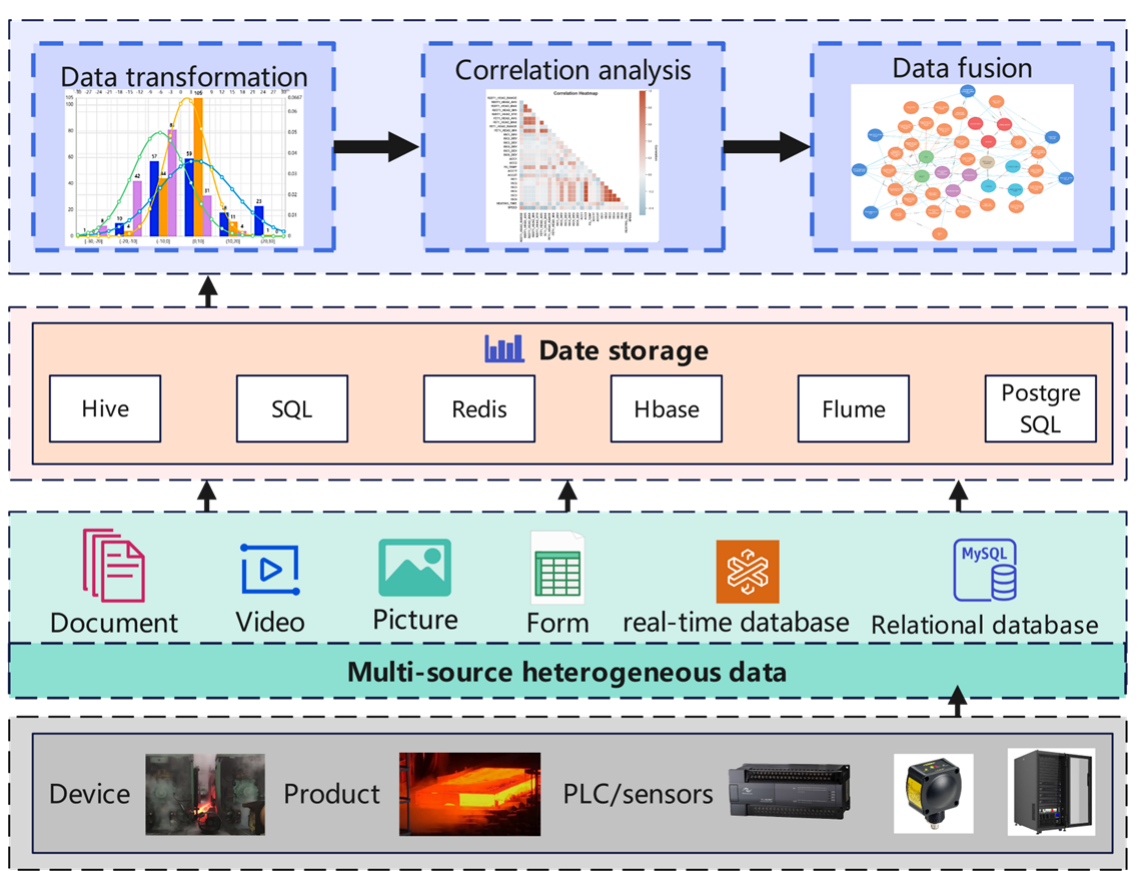
Data perception layer in hot-rolling platform.

The data collected by the entity perception layer are multisource heterogeneous data. The edge layer needs to perform data processing, including spatiotemporal transformation, data standardization, and data regeneration, on these data. As shown in Figure 2, protocol parsing middleware technology is compatible with Modbus, OPC, CAN, Profibus, and other protocols and adopts a software communication interface to achieve data format conversion and unification. The edge layer uses HTTP, MQTT, and other protocols to transfer data from the edge to the cloud to achieve remote data access and create a global data space for the collaborative design platform. All kinds of data processed by the edge layer are transmitted to the twin layer for further processing and display.

#### 2.1.3. Virtual Entity

The Virtual Entity is the core of the fault diagnosis platform, including the 3D model, mechanism model, finite element model, and data-driven model. The 3D model primarily serves as a roaming monitoring tool for the production line. The mechanism model is responsible for analyzing the mechanism of the field equipment and then determining the key parameters that affect the fault. The finite element model is employed to simulate the rolling process and generate simulated data. Data-driven models build black box or gray box models through the analysis of historical data to establish the relationship between the input and output and then perform tasks such as fault diagnosis and prediction.

#### 2.1.4. Application Layer

The application layer includes modules for equipment monitoring, fault alerts, and fault diagnosis. Frontline workers directly manipulate the application layer modules to coordinate the entire system, thereby enabling decision deployment at the entity perception layer.

### 2.2. Revising the Construction of a Diagnostic Model for Temperature Anomalies in Hot-Strip Final Rolling

We established a fault diagnosis model for the hot-strip final rolling temperature based on the mechanism and data. The head of the strip is more affected by the set value of the final rolling temperature model, and the body of the strip is mainly affected by the dynamic continuous regulation of the final rolling temperature; thus, in this study, different research methods are carried out according to the different data types of the strip head and body.

For the strip head data, the influencing factors and characteristic values of the head were selected according to the mechanism model, and the characteristic values were processed and standardized. The random forest model was used to screen the importance of the characteristic variables. The outlier score of the current sample was calculated with the isolation forestalgorithm, and the numerical risk assessment method was established. For the data in the middle of the strip, the abnormal section of the curve data was identified and eliminated, and normalization and length scaling were carried out. The outlier samples were screened with the Hausdorff distance, and the similarity between curves was calculated using the *LCSS* algorithm to establish a curve risk assessment method.

**Figure 3:**
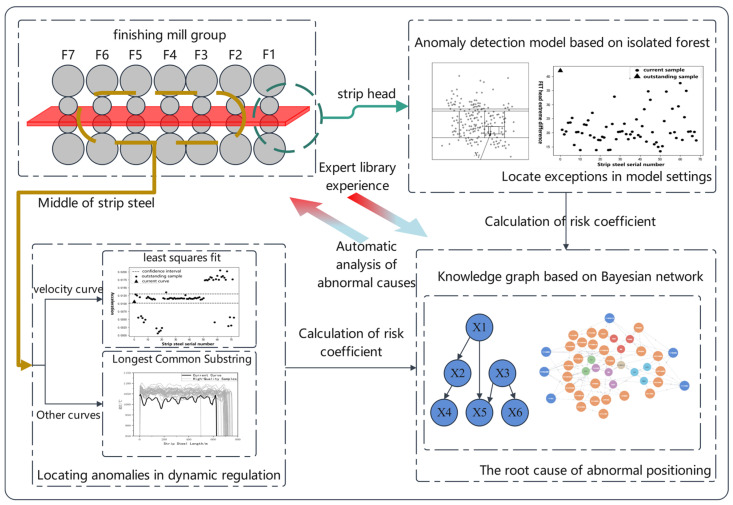
Resolution process flowchart for the abnormal final rolling temperature.

Finally, a knowledge map of the strip final rolling temperature anomaly diagnosis combined with a Bayesian network was constructed, and the coupling influence relationship between related variables was obtained according to the model mechanism knowledge. The risk coefficient obtained according to the risk assessment model of the strip head and body was searched and reasoned according to the node attributes in the knowledge map, and the root cause of the anomaly was finally traced. The technical route of this research is shown in Figure 3.

#### 2.2.1. Data Collection

The data for this study were obtained from the 2250 hot-rolling production line at Maanshan Iron & Steel Company Limited (Maanshan, China). Because the finishing rolling process and the target final rolling temperature of products with different steel grades are different, a single steel grade product was selected for analysis. Due to the small amount of data with exactly the same steel grade specifications, products with the same steel grade and specifications with a width of ±20 mm and a thickness of ±0.5 mm with no abnormal final rolling temperature were selected as excellent samples. A total of 100 excellent samples with a steel grade of MRTRG00502, a width range of [1682 mm, 1707 mm], and a thickness of 4 mm were selected, of which 68 were positive samples and 32 were negative samples, and the negative samples included the current strip steel to be analyzed.

**Figure 4:**
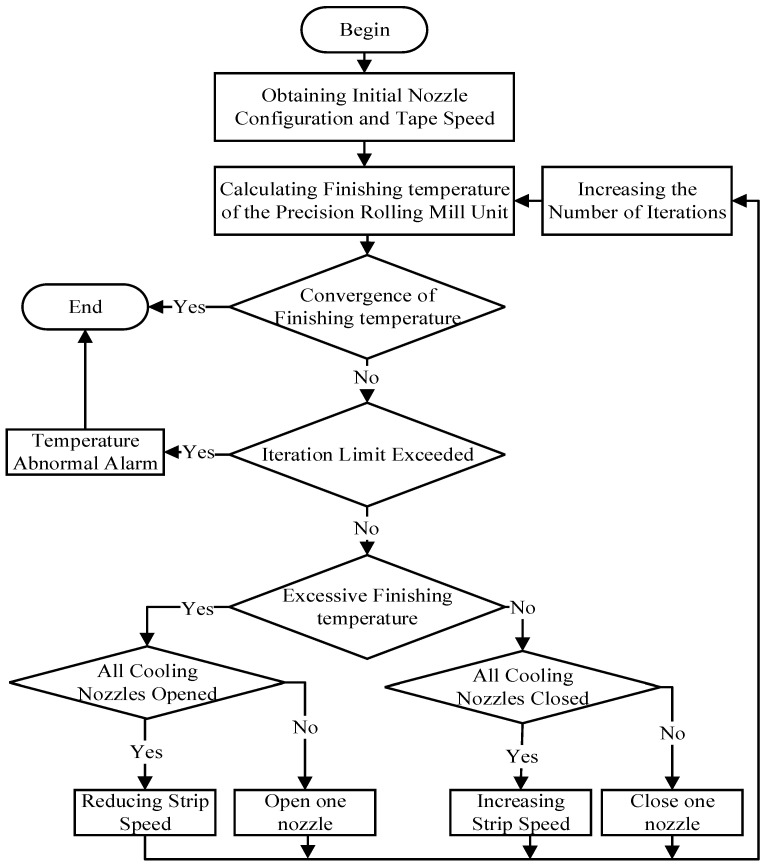
Final rolling temperature setting calculation model.

After the strip is perforated, its head temperature is mainly affected by the system setting model [13]. The setting model of finishing rolling calculates parameters such as the speed and opening water in the rolling process through the temperature at the finishing rolling entrance and the target finishing rolling temperature to ensure that the actual finishing rolling temperature can reach the target range [14]. The final rolling temperature setting model of the field system mainly included the initialization of the configuration parameters, the calculation of the time–velocity curve, the calculation of the predicted value of the final rolling temperature, and the self-learning module of the system. Through the coordination of these modules, the temperature of the strip head can be predicted and controlled to achieve the target temperature of the final rolling. Figure 4 shows the flow chart of the calculation model for setting the final rolling temperature [15].

As seen from the figure above, the cooling nozzle flow rate and strip threading speed can control the final rolling temperature, and the nozzle flow rate will be considered as an influencing factor in the follow up to this study. For the strip threading speed, the simplified temperature drop Formula (1) of the finishing mill group can be deduced [16]:

(1) $$\frac{t_{i}\;-t_{w}}{t_{FE0\;}-t_{w}}=\exp(\frac{-P_{r}\sum_{j=1}^{8}L_{j}}{h_{n}v_{n}}).$$

Using the simplified temperature drop Formula (1) of the finishing mill group, the final stand speed Formula (2) can be derived as follows:

(2) $$v_{n}=\frac{-P_{r}\sum_{j=1}^{8}L_{j}}{h_{n}\ln\left(\frac{T_{FC\;}-T_{W}}{T_{FEO}-T_{W}}\right)}.$$

Formulas (1) and (2) are the $t_{i}$ (°C) strip temperature for each frame, *i* = 1~7; $t_{FE0}$ (°C) is the finishing inlet temperature, and $t_{w}$ (°C) is the descending temperature drop. $L_{j}$ (m) for the distance between the frame, the frame segmentation of the high-temperature meter from the entrance to the export pyrometer, and the pyrometer distance between the F1 and F7 frame are also included, as well as *j* = 1~8; $h_{n}\;\left(mm\right),v_{n}\;\left(m/s\right)$ are the finishing exit thickness and exit velocity, respectively. $P_{r}$ (m/s) stands for the Prandtl number [17], and its specific calculation Formula (3) is as follows:

(3) $$p_{r}=\frac{2\alpha }{\gamma c},$$

where α (kg/°C × s^3^) stands for the equivalent cooling coefficient, γ (kg/m^3^) represents the density, and c (kcal/°C × kg) denotes the specific heat capacity.

According to Formulas (1) and (2), the parameters that affect the setting of the final rolling temperature mainly include the rolling speed, the finishing rolling inlet temperature, the finishing rolling outlet thickness, the equivalent cooling coefficient, the descaling temperature drop, and the distance between frames. The outlet thickness of finishing rolling is determined by the production target, and the descaling water and the distance between racks are fixed values; therefore, the parameters that can be controlled in rolling are only the rolling speed, the finishing rolling inlet temperature, and the equivalent cooling coefficient, which is determined by the cooling water flow rate between the stands [18].

The inlet temperature of the finishing rolling can be traced back to rough rolling, the heating furnace, and other processes, including the rough rolling outlet temperature (RDT), the oven temperature, and other process parameters [19], in addition to the data collected by the platform’s entity perception layer, combined with the actual situation of field production and the experience of technical personnel, as well as the analysis results from the aforementioned mechanistic models. The following parameters for setting the final rolling temperature model were selected, as shown in Table 1 and Table 2.

#### 2.2.2. Data Preprocessing

The different dimensions between various process parameters will have a greater impact on subsequent data dimensionality reduction and risk coefficient calculations. This issue can be addressed through normalization or standardization. After conducting research, it has been found that, in comparison to normalization, standardization does a better job of handling outliers. However, outliers are precisely what we need to study, so standardization is preferred for data processing due to its wider applicability [20].

Common standard methods include Max-Min normalization, Logistic normalization, and Z-Score standardization. Max-Min normalization, however, does not handle newly introduced outlier data well, while Logistic normalization assumes that the dataset is distributed around zero, which is not consistent with our research dataset. Therefore, we have chosen the Z-Score standardization method, which effectively eliminates the inconvenience caused by data with different magnitudes for data analysis and ensures comparability between the data points [21].

For data samples $\left(X_{1},X_{2},X_{3},\ldots ,X_{i}\right)$, Z-Score standardization transforms the data into a distribution with a mean of 0 and a standard deviation of 1 using the following formula:

(4) $$S=\frac{X_{i}-\mu }{\sigma },$$

where *S* is the standardized value, *X_i_* is the original data point, *μ* is the mean (average) of the data sample, and *σ* is the standard deviation of the data sample.

**Figure 5:**
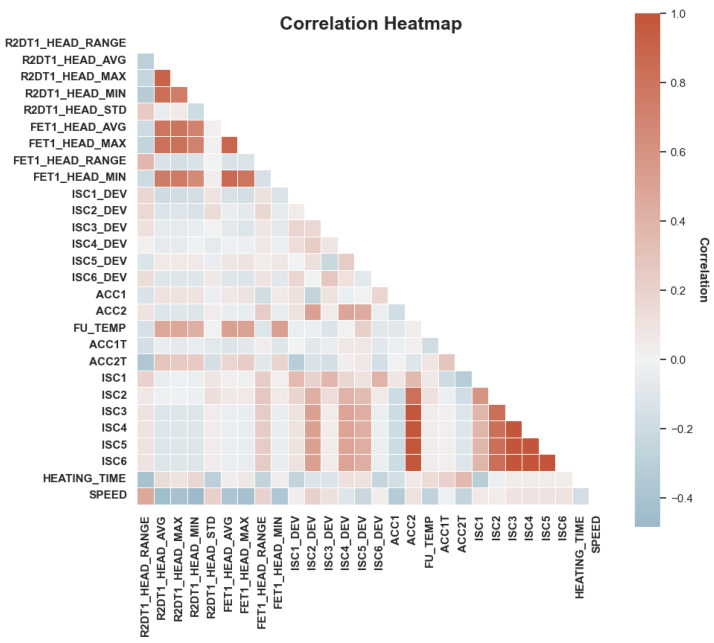
Feature correlation distribution.

Figure 5 depicts the feature correlation distribution diagram, which quantifies the degree of correlation among variables using a correlation matrix. Based on the results of correlation analysis, we can choose to eliminate certain redundant data with high correlation. For instance, there is a high degree of correlation between IS2 and IS3, IS4, and IS5, which constitutes redundant data. Excessive redundancy in the data can negatively impact the model’s accuracy, and therefore, it should be considered for removal. The system calculates the importance of all characteristic variables through random forest and sorts them according to the importance size. The optimization of parameters and the evaluation of the random forest model are performed using the ROC-AUC [22] metric. When n_estimators = 50 and max_depth = 2, AUC = 0.92 is the maximum value, indicating that the random forest model has a good effect. The optimal parameters selected above were introduced into the random forest model.

**Figure 6:**
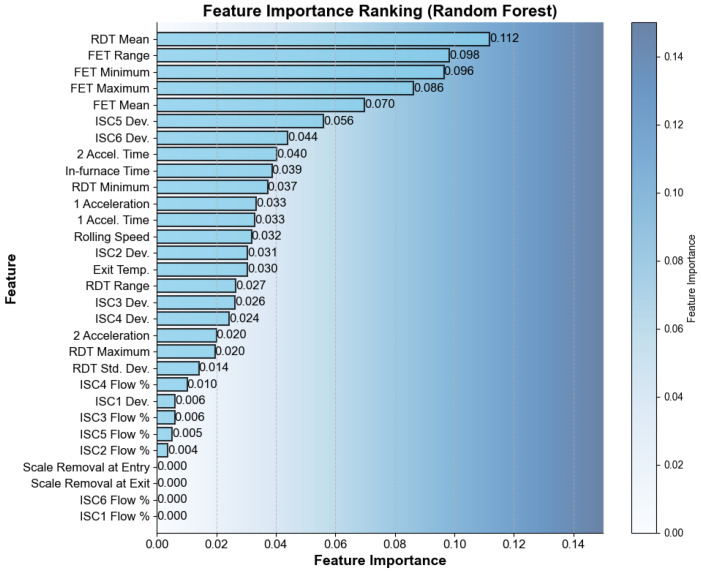
Feature importance distribution.

Figure 6 illustrates the distribution of the feature importance. According to the feature importance of each parameter, the importance of variables such as the F1–F7 interrack cooling water opening percentage, the two acceleration rate, and the dephosphorization switch at the inlet and outlet were significantly lower than other feature variables. Based on the figure, the variables were sequentially aggregated from the highest to lowest importance values. The variables with cumulative importance values within 0.9 were selected for further investigation.

**Figure 7:**
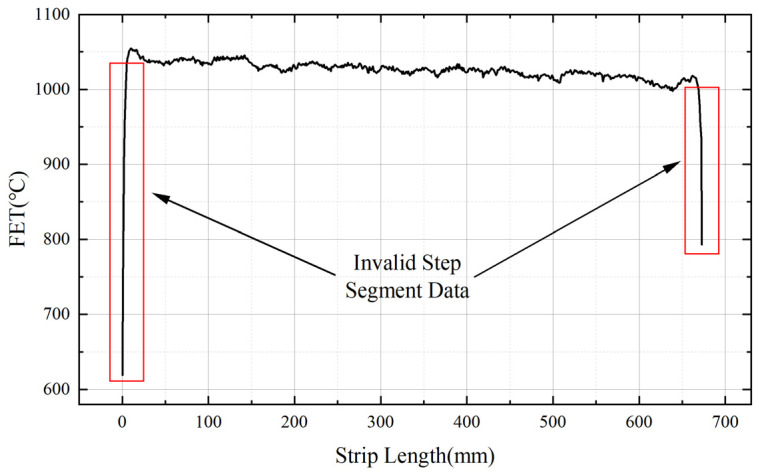
Strip steel final rolling temperature process curve.

When strip steel passes through the inspection instruments at the head and tail, it generates a step change in the data. This is due to the strip steel’s temperature being significantly higher than that of the conveyor rollers, resulting in a step change in temperature readings when the temperature sensor detects the strip steel. To ensure the accuracy of the subsequent data calculations, it is necessary to select the valid data for the entire length of the strip steel and eliminate the step change data at the head and tail. The strip process curve is shown in Figure 7.

The next step would be to calculate the length of the step change segment. Because the width change in the finishing rolling process has little effect on the final rolling temperature, the head and tail parts of the strip can be determined according to the thickness ratio of the finishing rolling inlet and outlet. The formula is as follows:

(5) $$L_{CH}=L_{CT}=\frac{h_{E}}{h_{T}},$$

where $L_{CH}$ (mm) and $L_{CT}\;$(mm) are the head length removed and tail length removed, respectively, $h_{E}$ (mm) is the measured thickness of the strip finishing inlet, and $h_{T}\;$(mm) is the target thickness of the strip finishing.

### 2.3. Model Establishment

#### 2.3.1. Establishment of the Strip Head Model

Combined with the actual onsite data, the abnormal samples of products per day were far smaller than the qualified samples, and the positive and negative samples were unbalanced, while the isolation forest was suitable for the situation where the proportion of negative samples was small. Therefore, the isolation forest method was used in the abnormal evaluation of the single-point values. The basic idea of the isolation forest (IF) algorithm is to separate the normal data from the abnormal data by constructing an isolated tree based on a random forest [23].

First, we build the isolated tree. Suppose we have a set of data $X=\left\{x_{1},x_{2},\ldots ,x_{n}\right\}$, including *n* samples, and each sample has *d* features, namely $\forall x_{i}\in X,x_{i}=\left(x_{i1},x_{i2},\ldots ,x_{id}\right)$. A dimension *q* is randomly selected from *d* dimensional features, and a cutting point *p* is randomly generated in the current data.

(6) $$\min(x_{ij},j=q,x_{ij}\in X^{\prime })<p<\max(x_{ij},j=q,x_{ij}\in X^{\prime })$$

The $X^{\prime }$ is a subset of *X*; for each sample point $x_{i}$, it can be calculated in each tree path length $h\left(x_{i}\right)$ to determine whether it is an abnormal point. To evaluate the abnormal scores, the abnormal score computation formula is as follows:

(7) $$s\left(x,\psi \right)=2^{\frac{E\left(h\left(x\right)\right)}{c\left(\psi \right)}},$$

(8) $$H\left(i\right)=\ln(i)+\gamma ,$$

(9) $$c\left(\psi \right)=\left\{\begin{array}{c} 2H\left(\psi -1\right)-\frac{2\left(\psi -1\right)}{\psi }\\ 1\\ 0 \end{array}\right.\begin{array}{c} \left(\psi >2\right)\\ \left(\psi =2\right)\\ \left(otherwise\right) \end{array},$$

where $E\left(h\left(x\right)\right)$ is the average path length of the sample in all isolated trees, $c\left(\psi \right)$ is the average path length of the isolated tree for the dataset with sample *n*, and *γ* = 0.5772156649 is the Euler constant. Thus, the construction of the isolation forest algorithm model for the head single-point value of strip steel is now complete.

#### 2.3.2. Establishment of the Strip Steel Body Model

Due to the variable curve anomalies, this study constructed an average curve based on sample data selection and used the Hausdorff distance to screen samples with a large deviation degree. Then, the *LCSS* algorithm was used to evaluate the curve similarity between the current curve and excellent samples and establish a risk assessment model based on curve similarity.

The Hausdorff distance is the distance between two points set measuring [24] and is equipped with two sets of collection: $A=\left\{a_{1},a_{2},\ldots ,a_{i}\right\},\;\;B=\left\{b_{1},b_{2},\ldots ,b_{j}\right\}$. The Hausdorff distance between the two sets is as follows:

(10) $$\left\{\begin{array}{c} H\left(A,B\right)=\max(h\left(A,B\right),h\left(B,A\right))\\ h\left(A,B\right)=\max_{a_{i}\in A}(\min_{b_{j}\in B}(dist(a_{i},b_{j})))\\ h\left(B,A\right)=\min_{b_{j}\in B}(\max_{a_{i}\in A}(dist(b_{j},a_{i}))) \end{array}\right.,$$

where $dist\left(a_{i},b_{j}\right)$ is the $a_{i}$ and $b_{j}$ Euclidean distance and $H\left(A,B\right)$ for the two-way Hausdorff distance; after screening, the Hausdorff distance sample curve was more concentrated, which is helpful to improve the accuracy of the risk assessment model. The average value of each point is selected according to the excellent sample curve, and a new average curve is constructed to represent the average level of the excellent sample curve.

**Figure 8:**
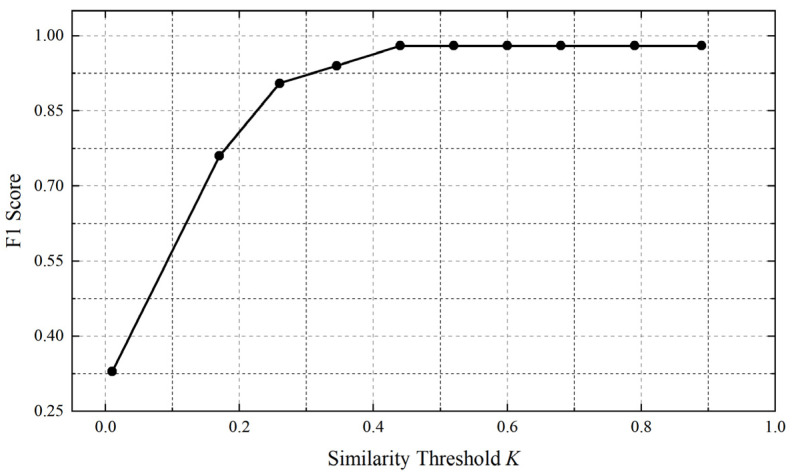
Flow chart for predicting the health of the back-up roll using the Copula function.

The longest common substring (*LCSS*) algorithm can measure the similarity between two curves by finding the longest common substring between them [25]. Given two time series datasets *A* and *B*, the lengths of which are *n* and *m*, respectively, the longest common subsequence length is found as follows:

(11) $$LCSS=\left\{\begin{array}{c} 0\\ 1+LCSS\left(a_{t-1},b_{i-1}\right)\\ \max(LCSS\left(a_{t-1},b_{i}\right),LCSS\left(a_{t},b_{i-1}\right)) \end{array}\right.\begin{array}{c} \left(A=\emptyset ,B=\emptyset \right)\\ \left(dist\left(a_{t},b_{i}\right)<k\right)\\ \left(otherwise\right) \end{array},$$

including t∈n,  i∈m, the similarity threshold *k* for members, $dist\left(a_{t},b_{i}\right)$ for its members, and the Euclidean distance between parallel when the distance between the members is less than the threshold similar to two members. The greater the *LCSS* value is, the greater the similarity between the two curves; in contrast, the difference between the two curves can be obtained. Then, the risk coefficient can be calculated by the following formula $Risk_{c}$:

(12) $$Risk_{c}=\left(1-\frac{LCSS\left(A,B\right)}{\min(n,m)}\right),$$

including $Risk_{c}$, the risk coefficient for the curve, $LCSS\left(A,B\right)$ for the current curve and the curve of the average *LCSS*, and min(n,m) for two curves of the shortest length. Excellent samples after the Hausdorff distance selection are all samples that are very close to the mean curve. This part of the samples is used as a training set to train the *k* value of the *LCSS* model, and then the similarity between the current curve to be measured and the mean curve is returned. We calculate the Hausdorff distance between all excellent samples and the average curve, calculate the *LCSS* similarity between the RDT curve and the median curve of each sample, and take different *k* values to calculate the F1 value during calculation. With the continuous increase in the *k* value, the F1 value also gradually rises. When the *k* value exceeds the maximum difference between two curves, it is considered that all points of the two curves are similar. At this time, if the *k* value continues to increase, the F1 value will not significantly improve, and it is considered that the *k* value at the inflection point is the most appropriate. For example, as shown in Figure 8, the F1 value of the excellent sample and the median curve are the most appropriate threshold values when *k* = 0.4 is taken:

The calculation of the RDT similarity threshold for all samples is displayed in Table 3. The next step is to select the best *k* value, which is based on the maximum distance between the sample and the average curve. The sample curve may not be all risk-free, allowing a certain range of fluctuations. Some samples that are close to the median curve have small *k* values, while those that are far away from the mean will have larger *k* values. Taking the average of the best *k* values of all samples can balance the problem of *k* values that are too small or too large. So we take the average: $\bar{k}=0.43$, this value is used as the RDT similarity threshold of the final member of the *LCSS* model.

#### 2.3.3. Establishment of a Comprehensive Prediction Model for Strip Steel

**Figure 9:**
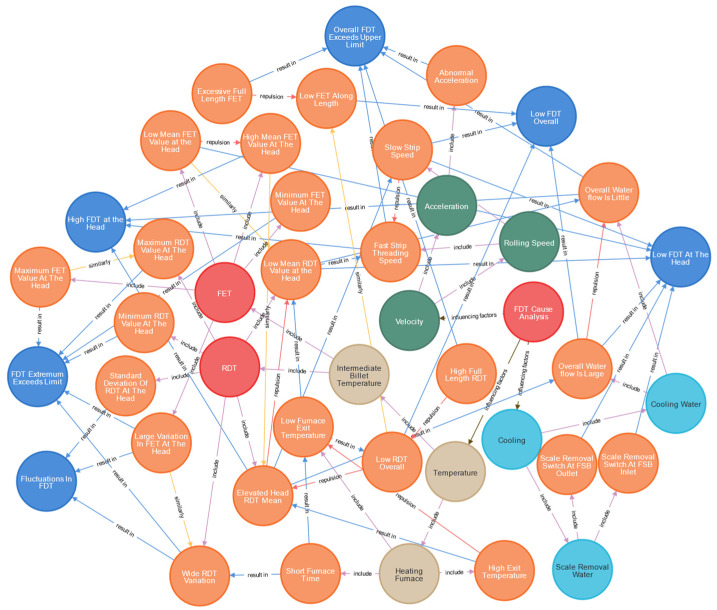
Knowledge graph model for abnormal final rolling temperature diagnosis.

Since the knowledge graph can model the process data of production and the relationship between the organization and can be visualized in the form of a graph, it is very suitable for the coupling effect between the parameters of the final rolling temperature process in this study [26]. At present, there are three kinds of storage methods for knowledge graphs: RDF storage, relational database storage, and graph database storage. Compared with relational databases and RDF, graph data are more suitable for processing connected data, more flexible data modeling, and better scalability and fault tolerance [27]. Therefore, this paper adopts the Neo4j graph database for anomaly inference. According to the research results in the previous two sections, the influential factors, extracted feature variables, and the influence relationship between them were added to the graph database Neo4j, and the relationship between nodes was established in combination with the field quality ledger experience. A total of 85 relationships were established, and after the nodes and relationships of the knowledge graph of the final rolling temperature anomaly analysis were constructed, the abnormal causes could be reasoned and diagnosed using the knowledge reasoning method. As shown in Figure 9, according to the attribute judgment and relationship of each node, a complete cause path of the final rolling temperature anomaly was finally obtained. While mining knowledge nodes and relationships, a knowledge graph can also be customized according to the actual situation of the site to achieve a customized analysis of the site.

**Figure 10:**
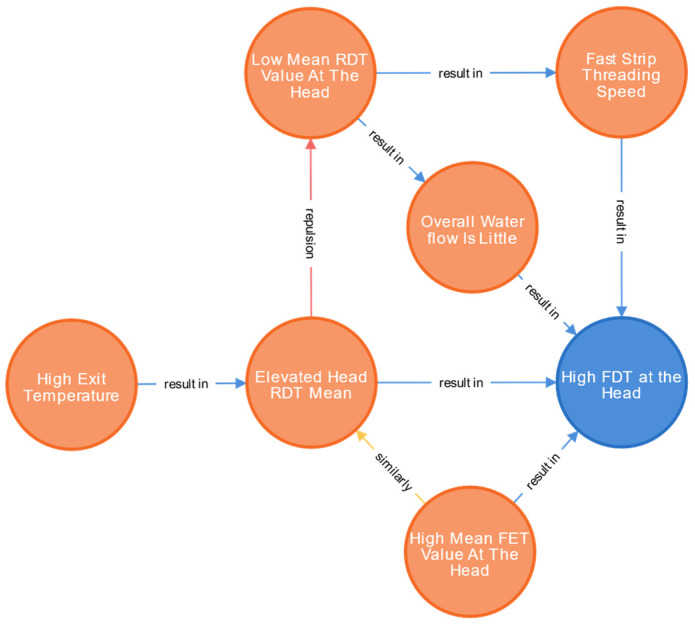
The influence of coupling relationship between “FDT head higher” nodes.

Figure 10 is a partial relation node in Figure 9. The colour of the node itself has no special meaning, it is just for aesthetics. There are different relationships between nodes. We use different colours and words to distinguish them. Blue stands for "result in", red for "repulsion" and yellow for "similar", pink for "include" and brown for "influencing factors". It can be seen that the direct nodes that cause “high FDT head” include “fast tape penetration speed”, “low overall open water volume”, “high average RDT head”, and “high average FET head”. Since there is no other process between the roughing outlet and the finishing inlet, only the thermal insulation roller table is passed, and it can be considered that the temperatures of the RDT and FET are similar. The correlation node of “high RDT head mean” is “high oven temperature”, and a high oven temperature will cause a high roughing outlet temperature. The relevant nodes of the two nodes of “low overall opening water” and “fast threading speed” are both caused by a “low RDT head mean”, which may be due to a low RDT temperature resulting in a less calculated opening water and a fast threading speed. When “high RDT head mean” and “low total open water” or “low ribbon speed” occur at the same time, the latter two nodes will find the relevant node “low RDT head mean”, and the nodes “low RDT head mean” and “high RDT head mean” contradict each other. Therefore, when the exclusive node is found at the same time, the node closest to the abnormal performance prevails.

Anomaly inference through a knowledge graph is based on the identification of anomalies at each node. However, this paper calculates the risk coefficient of each influencing factor and does not determine a clear threshold to indicate the abnormal risk coefficient. Therefore, this paper uses a Bayesian network for further anomaly inference, which can represent the probabilistic relationship between each variable [28]. This study combines a knowledge graph and a Bayesian network to carry out probabilistic reasoning to obtain the root cause of a parameter anomaly.

## 3. Experimental Results

### 3.1. Strip Head Risk Assessment Results

Based on the content of Section 2.3.1, an anomaly detection model was constructed using the isolation forest algorithm to diagnose anomalies in the head portion of strip steel. The analysis was conducted on a total of 69 coils of strip steel, which included both the current research samples and the excellent samples. The abnormal scores for the head portion’s RDT range are depicted in Figure 10.

**Figure 11:**
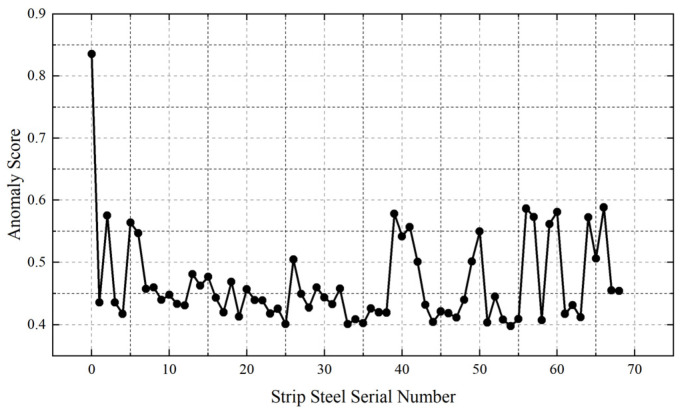
Scatter plot of RDT range abnormality scores.

The first point in Figure 11 represents the anomaly score for the current strip steel. The anomaly score for the current strip steel was 0.83, significantly higher than that of the other samples, indicating a notably elevated level of abnormality. Most of the remaining samples were distributed within the range of [0.4, 0.6], suggesting stability in the rolling process for this subset, with the RDT fluctuating within a defined range.

It is worth noting that, even among the excellent samples, there may be instances of higher anomaly scores for individual process parameters. However, these elevated scores may be due to anomalies in specific process parameters; through coordinated adjustments in other process parameters, the final strip steel temperature remained within acceptable limits, hence its inclusion in the excellent sample database.

For excellent samples, there are also points with high abnormal scores, which may be abnormal for a single process parameter, but through the coordination and adjustment of other process parameters, the final rolling temperature qualification of the strip steel eventually appears in the excellent sample bank. The purpose of establishing this anomaly detection model is to determine whether the process parameters of the current strip steel are abnormal. The isolation forest anomaly scores for other process parameters of the currently analyzed strip steel can be found in Table 4. It can be seen that the serious anomaly was the range of the RDT and FET headers, and the original data distribution can also prove that the two process parameters were abnormally large.

### 3.2. Strip Body Risk Assessment Results

The RDT current curve was obtained using the *LCSS* algorithm, and the sample average curve of the longest common subsequence length 179 participated in the calculation length of a total of 549 points, calculated according to the current strip type 10 for the risk coefficient of the RDT curve $Risk_{c}=67.39\%$. The rest of the process curve of the calculation parameters is shown in Table 5. The risk coefficient of the RDT and FET curves was higher than that of the other process curves for the steel coils to be tested.

**Figure 12:**
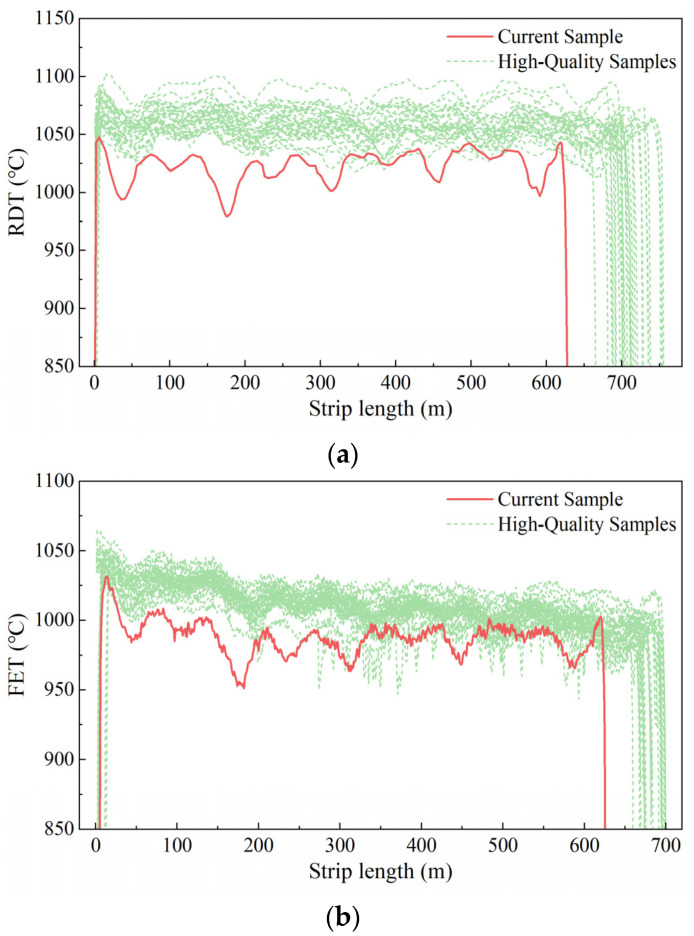
Original curves of RDT and FET; (**a**) Original curves of RDT; (**b**) Original curves of FET.

Based on the original data from the RDT curve (Figure 12a) and FET curve (Figure 12b), it is evident that the current curve to be measured deviated from the overall excellent sample to a large extent.

### 3.3. Comprehensive Root Cause Analysis Model of Strip Steel

**Figure 13:**
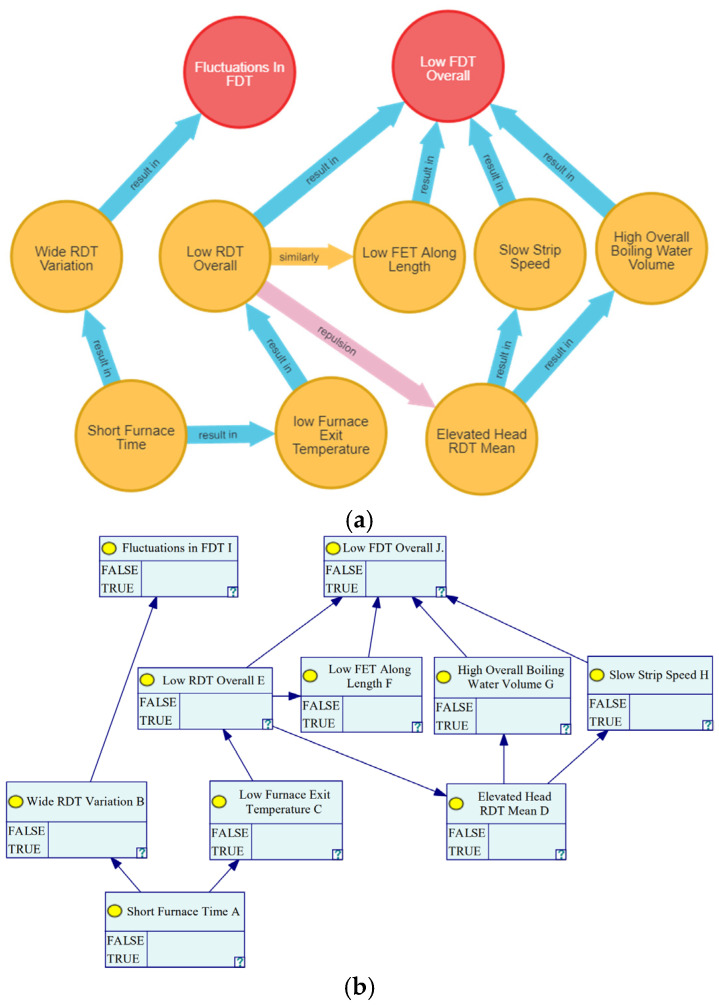
Abnormal final rolling temperature diagnosis graph and its transformed Bayesian network model diagram; (**a**) Abnormal final rolling temperature diagnosis graph; (**b**) Bayesian network model diagram.

Based on the on-site assessment of the final rolling temperatures, it is apparent that the final rolling temperature of the anomalous samples exhibited an overall lower tendency, coupled with multiple localized extreme points. Utilizing the nodes associated with FDT anomaly types, we identified two critical nodes: “FDT Fluctuation” and “Overall Low FDT”. Subsequently, this culminates in the creation of the diagnostic subgraph for final rolling temperature anomalies, as illustrated in Figure 13a. Following this, we proceeded to establish a Bayesian network model, as depicted in Figure 13b, within GeNIe. GeNIe, characterized as a graphical user interface-based Bayesian network modeling tool, is instrumental in constructing, analyzing, and inferring Bayesian networks [29].

The Bayesian network needs to input the conditional probability table of each node. According to the production volume of the site for a week, 70 coils of strip steel with abnormal final rolling temperatures were selected as samples from the quality analysis ledger to calculate the conditional probability of each node. The specific analysis contents of the ledger are shown in Table 6.

**Figure 14:**
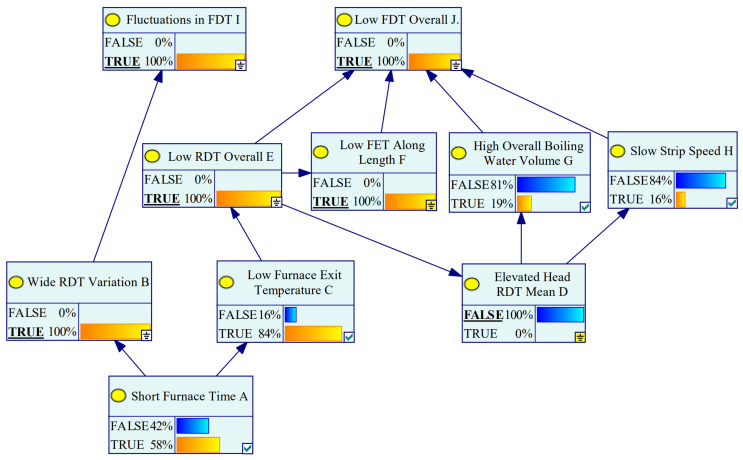
Bayesian network diagnosis results.

According to statistics, the probability of each node occurring independently is shown in Table 7:

With the conditional probability distribution of each node, the probability of the occurrence of uncertain nodes can be deduced according to the posterior probability of node updating to find the root cause of the final rolling temperature. According to the risk coefficient calculated in the aforementioned research, the influencing factors with risk coefficients greater than 50 are shown in Table 8. The status of such nodes is set to “TRUE” to indicate that anomalies were identified, and the Bayesian network is updated, as shown in Figure 14.

As shown in Figure 14, according to the calculation of the Bayesian network, when the two anomalies of “FDT fluctuation” and “overall low FDT” are known to have occurred, the probability of the “short furnace time” node occurring is 58%, and the probability of the “low oven temperature” node occurring is 84%, indicating a large range of incoming material temperatures. The uneven temperature is probably caused by the short time in the furnace. By observing the furnace time and temperature of the current sample, it was found that the sample was indeed at a low level. By comparing with the actual parameter diagram, it is known that the overall low FDT was due to the low incoming RDT temperature, while the rolling speed and the opening of the cooling water between racks were opened according to the normal RDT temperature. The result was a low FDT. Due to the existence of an in-furnace time close to that of the current sample in the excellent sample, no obvious abnormality of the in-furnace time of the current sample was found in the single-point risk coefficient assessment in Section 3, and the subsequent anomaly analysis revealed that the in-furnace time of the current sample was not suitable for this slab. In other words, it was successfully found that the root cause of the final rolling temperature anomaly was the lack of time in the heating furnace.

Finally, the model is verified on site. Due to the absence of clear standards for anomaly diagnosis and the fact that the knowledge graph database in the model was constructed based on expert experience, the method used to validate the accuracy of this model involved comparing the anomaly causes identified by the model with those identified by technicians.

The automatic analysis model for the abnormal final rolling temperature was configured with process parameters and a knowledge graph that consolidates diverse experiential knowledge. As a result, it provided a more comprehensive analysis compared to individual technicians. During on-site inspections, the accuracy was determined via the agreement between the causes identified by the model and those identified by the technicians. Following the application of this research in the field, 50 samples of abnormal quality for each quality analysis item were selected, totaling 400 samples for comparison. The specific accuracy of the automatic analysis of final rolling temperature, the focus of this study, is presented in Table 9.

The analysis accuracy exceeded the project contract requirement of 90%, with the model achieving an accuracy rate of 92%, meeting the on-site requirements and successfully passing the acceptance test.

## 4. Conclusions

In this study, a fault diagnosis platform for hot-rolled strips based on a digital twin is established, and the fault diagnosis model of the final rolling temperature of the strip is established based on the platform. The random forest algorithm was used to analyze the importance of 30-dimensional process parameters in 100 coils of strip steel on site, and standardization processing was carried out to reduce the data dimension to 18 dimensions. The isolated forest algorithm was used to monitor the abnormal process parameters of the strip head data, and the *LCSS* curve similarity algorithm was used to build a risk assessment model for the strip body curve data. Finally, according to the mechanistic relationship between the process parameters and expert experience, a knowledge graph is established, and a Bayesian network is used for automatic inference analysis. Based on a practical case, it is found that the root cause of the abnormal final rolling temperature of the target steel coil is the lack of time in the heating furnace. After running in the steel mill production line for one month, when compared to the manual analysis results, the automated analytical model developed in this research achieves an accuracy rate of 92%, meeting the precision requirements of the actual steel mill.

This project, based on the understanding of the influencing mechanisms of the hot-rolling exit temperature and combined with expert knowledge, has developed an automated analytical model using relevant data-driven algorithms. This model was deployed and demonstrated its effectiveness in practical production settings. However, there are still areas that can be further refined and improved:

1. In addition to investigating the process parameters that affect the exit temperature at the hot-rolling control level, future research can delve deeper into the material-specific micro-mechanisms of strip steel. This can help enhance the knowledge graph and provide a stronger theoretical foundation for automated analysis.
2. In terms of data-driven approaches, the incorporation of additional data mining and machine learning techniques can enhance the precision of analysis and the efficiency of the algorithm’s execution.

## Figures and Tables

**Table 1:** Strip steel head final rolling temperature process parameter table.

Process Parameter Category	Serial Number	Process Parameter Description
Incoming Material, Temperature-Related	1–12	RDT Head (°C): Range, Mean, Maximum, Minimum, Standard Deviation. FET Head (°C): Range, Mean, Maximum, Minimum, Standard Deviation. Exit Temperature (°C): In-Furnace Time (s).
Rolling Process, Cooling-Water-Related	13–26	ISC1-6 (m^3^/h): Setpoint Deviation, Flow Percentage. Finishing Mill Exit Descaling Switch. Finishing Mill Entry Descaling Switch.
Rolling Speed-Related	27–31	Rolling Speed (m/s), Acceleration 1 (m/s^2^), Acceleration 2 (m/s^2^), Acceleration Time 1 (s), Acceleration Time 2 (s).

**Table 2:** Strip steel body final rolling temperature process curve table.

Process Curve Name	Unit	Process Curve Description
RDT	°C	Roughing Delivery Temperature
FET	°C	Finishing Entry Temperature
F7_SPEED	m/s	F7 Rolling Speed
ISC1	m^3^/h	1, 2 Interstand Cooling Water
ISC2	m^3^/h	2, 3 Interstand Cooling Water
ISC3	m^3^/h	3, 4 Interstand Cooling Water
ISC4	m^3^/h	4, 5 Interstand Cooling Water
ISC5	m^3^/h	5, 6 Interstand Cooling Water
ISC6	m^3^/h	6, 7 Interstand Cooling Water

**Table 3:** Calculation results of the member similarity threshold.

Sample Number	Member Similarity Threshold *k*
1	0.4
2	0.4
3	0.4
4	0.4
5	0.3
6	0.4
…	…
66	0.4
67	0.3
68	0.4

**Table 4:** Isolation forest anomaly score table.

Process Parameters	Isolation Forest Anomaly Score
RDT Head Range	0.83
FET Head Range	0.76
In-Furnace Time	0.57
Exit Temperature	0.56
FET Head Mean	0.53
FET Head Maximum Value	0.51
ISC1 Deviation Between Setpoint and Actual Value	0.51
RDT Head Mean	0.50
RDT Head Minimum Value	0.48
…	…

**Table 5:** Process curve risk coefficient.

Feature Variables	Member Similarity Threshold k	*LCSS* Length	Risk Coefficient/%
RDT	0.43	179	67.39
FET	0.46	249	57.58
ISC4	0.51	380	40.91
ISC5	0.63	385	40.23
ISC6	0.50	447	30.59
ISC2	0.42	457	28.99
ISC3	0.43	461	28.45
ISC1	0.34	500	22.33

**Table 6:** Quality analysis ledger for abnormal final rolling temperature.

Serial Number	Steel Grade	Width	Anomaly Type	Anomaly Cause
1	MRTRG00502	1692	FDT Below Lower Limit: FDT Fluctuation	Low Exiting Temperature
2	MRTRG00502	1692	FDT Exceeding Upper Limit: FDT Fluctuation	High Strip Speed, Insufficient Cooling Water Flow
3	MRTRG00502	1699	FDT Below Lower Limit: FDT Fluctuation	RDT, FET Low Head Temperature, Maximum Strip Speed Reached with No Adjustment Margin
4	MRTRG00502	1699	FDT Exceeding Upper Limit: FDT Fluctuation	Low Cooling Water Flow, Maximum Strip Speed Reached
...	...	...	...	...

**Table 7:** Individual node occurrence probability table.

Node Name	Individual Node Occurrence Probability P
Short In-Furnace Time (A)	0.20
Low Exit Temperature (C)	0.30
RDT Large Range (B)	0.80
RDT High Head Mean (D)	0.84
RDT Overall Low (E)	0.16
FET Overall Length Low (F)	0.16
Overall Excessive Water Volume (G)	0.83
Slow Strip Speed (H)	0.67
FDT Fluctuation (I)	0.09
FDT Overall Low (J)	0.47

**Table 8:** Abnormal feature variables and risk coefficients.

Feature Variables	Risk Coefficient/%
RDT Head Range	66.45
FET Head Range	52.95
RDT Curve	67.39
FET Curve	57.58

**Table 9:** Quality analysis accuracy assessment form.

Quality Analysis Items	Total Number of Rolls in the Samples	Number of Accurate Rolls	Number of Unaccepted Rolls	Accuracy
FDT	50	46	4	92%

## Data Availability

The datasets generated during and/or analyzed during the current study are not publicly available due to the sensitive nature of the industry collaboration, but are available from the corresponding author on reasonable request.
